# Comparing proxy rated quality of life of people living with dementia in care homes

**DOI:** 10.1017/S0033291718003987

**Published:** 2019-01-29

**Authors:** S. Robertson, C. Cooper, J. Hoe, K. Lord, P. Rapaport, L. Marston, S. Cousins, C. G. Lyketsos, G. Livingston

**Affiliations:** Division of Psychiatry, University College London, Maple House, 149 Tottenham Court Road, London, W1T 7NF, UK

**Keywords:** Care home, carer, dementia, family carer, institution, paid carer, proxy, quality of life

## Abstract

**Background:**

Improving quality of life (QOL) for people with dementia is a priority. In care homes, we often rely on proxy ratings from staff and family but we do not know if, or how, they differ in care homes.

**Methods:**

We compared 1056 pairs of staff and family DEMQOL-Proxy ratings from 86 care homes across England. We explored factors associated with ratings quantitatively using multilevel modelling and, qualitatively, through thematic analysis of 12 staff and 12 relative interviews.

**Results:**

Staff and family ratings were weakly correlated (*ρ*_s_ = 0.35). Median staff scores were higher than family's (104 *v.* 101; *p* < 0.001). Family were more likely than staff to rate resident QOL as ‘Poor’ (χ^2^ = 55.91, *p* < 0.001). Staff and family rated QOL higher when residents had fewer neuropsychiatric symptoms and severe dementia. Staff rated QOL higher in homes with lower staff:resident ratios and when staff were native English speakers. Family rated QOL higher when the resident had spent longer living in the care home and was a native English. Spouses rated residents’ QOL higher than other relatives. Qualitative results suggest differences arise because staff felt good care provided high QOL but families compared the present to the past. Family judgements centre on loss and are complicated by decisions about care home placement and their understandings of dementia.

**Conclusion:**

Proxy reports differ systematically between staff and family. Reports are influenced by the rater:staff and family may conceptualise QOL differently.

## Introduction

Global dementia care strategies seek to enable citizens to live well with dementia (O'Rourke *et al*., 2015). Measuring quality of life (QOL) is one way to determine whether this aim is achieved. QOL is usually conceptualised as a broad, holistic construct (O'Rourke *et al*., 2015) representing how ‘good’ a person's life is overall (Livingston *et al*., 2014a). It is an important outcome for people with dementia as the illness is chronic and progressive. Interventions may reduce symptoms but also negatively impact QOL. In dementia, there may be no simple association between health-related QOL and an easily measurable clinical variable (Banerjee *et al*., 2009). Researchers are actively seeking ways to meaningfully measure QOL in dementia (Chua *et al*., 2016).

QOL, at least in part, is subjective and, therefore, ideally reported by the individual concerned. Care home residents have, in general, more severe dementia than people living with dementia in the community (Beerens *et al*., 2014) and many are unable to self-report QOL (Hoe *et al*., 2006). Often proxy reports are the only possible source of ratings (Magaziner, 1997; Hoe *et al*., 2006).

Where this is the case, the question of who provides these ratings and how this might influence the results must be considered (Graske *et al*., 2012). For research findings, interventions and policy to be meaningful, we need to understand differing proxy's views in proxy rated QOL and what proxy reports are measuring (Robertson *et al*., 2017). It may be that the evaluated success of an intervention in improving QOL depends on the perspective gathered (Goyder *et al*., 2012) and that family relatives and care staff perceive intervention effects differently (Clare *et al*., 2014). Understanding more about this complex outcome could also provide further targets for interventions to improve both perceived and actual QOL. In our recent systematic review, family and staff ratings were weakly correlated and different factors were associated with their reports. However, staff and family ratings have not been formally compared (Robertson *et al*., 2017).

For the first time, we investigated whether there is a difference in how paid staff and family members rate the QOL of care home residents with dementia using the DEMQOL-Proxy. We also explored using quantitative and qualitative methodologies, what influenced staff and family proxy ratings in the largest English national care home study to date.

## Methods

### Setting and sampling

This study is nested in the Managing Agitation and Raising QUality of LifE (MARQUE) national care home survey in England, full details and methods are published elsewhere (Livingston *et al*., 2017). MARQUE received ethical approval from the London (Harrow) NRES Committee (14/LO/0034). We recruited care homes from across England, ensuring inclusion of diverse provider type (voluntary, state, private), care provision (nursing or residential) and geographic (urban/suburban, rural) locations. We defined care home clusters as units within care homes in which staff and managers worked independently of all other units.

### Procedures

#### Residents

All residents with dementia were eligible for the study. We included all those with a diagnosis of dementia in their medical records. As many residents living with dementia will not receive a diagnosis (Challis *et al*., 2000) we screened remaining residents for probable dementia using the Noticeable Problems Checklist (NPC) (Levin, 1989) which has been validated against clinical diagnosis (Levin, 1989; Moriarty and Webb, 2000). Where staff identified residents as having capacity to agree to the study, they approached the residents first to ask if they agreed to talk to researchers about the project. If residents agreed, researchers trained to assess capacity made judgements using the Mental Capacity Act (2005) criteria. In most cases, people lacked this capacity, and we consulted the family carer. When there was no family carer available we sought a professional consultee, who knew the resident well.

#### Relatives

Relatives that visited the resident most often were approached by care home staff for agreement to be contacted by researchers. Researchers then contacted those who agreed, mailed information sheets and arranged a meeting to obtain informed consent in their preferred location.

#### Staff

Paid carers involved in the hands-on care of consented residents during the day, completed proxy measures with a research assistant. Staff consented to provide information about themselves.

#### Qualitative interviews

Qualitative interviews and analysis were undertaken prior to completing quantitative analysis to prevent bias. SR contacted staff and relatives participating in the MARQUE study who had provided proxy ratings of QOL to ask for their consent to take part in additional individual semi-structured interviews to explore their decisions about rating QOL. This interview was conducted separately after they completed the DEMQOL-Proxy questionnaire at a different time point. SR purposively recruited a maximum variation sample to cover the range of opinions (differing age groups, either sex, different roles in care homes or relationships) and continued interviewing until theoretical saturation was reached. The interview focused on their rationale for their choice of global rating on the DEMQOL-Proxy, either ‘Very Good, Good, Fair or Poor’. SR conducted interviews in a location chosen by the participant, either in their own home, a private room at UCL or the care home. SR remained flexible and open to emerging narratives. The length of interviews varied between 30 and 60 min.

### Measures

#### Care home

We recorded care home characteristics, including size; whether the home was residential or nursing; and any specialism. We completed an environmental survey: The Therapeutic Environment Screening Survey for Nursing Homes (TESS-NH) (Sloane *et al*., 2002). This observational instrument assesses different domains, including exit control; maintenance; cleanliness; safety; orientation/cueing; privacy; outdoor access; lighting; noise; visual and tactile stimulation; space and seating.

#### Resident

(1) *Quality of life:* The DEMQOL has psychometric properties that are at least as good as other QOL measures (Smith *et al*., 2007; Perales *et al*., 2013). The DEMQOL proxy is a 31-item version for paid staff and family carers; it is appropriate for use in mild, moderate and severe dementia (Smith *et al*., 2005). Higher scores indicate better QOL. Possible scores range from 31 to 124. The DEMQOL-Proxy provides a total score and a global rating ranging from ‘Very Good’, ‘Good’, ‘Fair’ or ‘Poor’.
(2) *Dementia severity*: The Clinical Dementia Rating (CDR) is a reliable, valid and widely used measure of global dementia severity (Hughes *et al*., 1982). It has a five-point scale summarizing information across six domains: Memory, Orientation, Judgment and Problem Solving, Community Affairs, Home and Hobbies, and Personal Care.
(3) *Neuropsychiatric symptoms*: The Neuropsychiatric Inventory (NPI) (Cummings *et al*., 1994) assesses 12 domains over the last 4 weeks: hallucinations; delusions; agitation/aggression; dysphoria/depression; anxiety; irritability; disinhibition; euphoria; apathy; aberrant motor behaviours; sleep and night-time behaviour; appetite and eating change. Each 12-point domain score is its frequency score multiplied by its severity score and the total is a sum of domain scores. Higher scores indicate greater severity.
(4) *Agitation*: The Cohen-Mansfield Agitation Inventory (CMAI) (Cohen-Mansfield and Billig, 1986) is a 29-item scale that systematically assesses agitation over the last 2 weeks. Each item rates a specific behaviour and its frequency. Possible scores range from 29 to 203. A score >45 indicates clinically significant agitation (Billig, 1986).

#### Staff

We recorded sex, ethnicity, years of experience and first language, whether they had a nursing qualification and their usual shift pattern (day or night shifts or mixed).

#### Family carers

We recorded age, sex, relationship to the person with dementia and how often they visited the person with dementia.

### Analysis

All quantitative analyses were completed in Stata 14 (StataCorp, 2015) with full datasets.

To investigate whether there is a difference in proxy ratings of QOL we:

(1) Calculated the correlation (Spearman's) between total scores.
(2) Compared ranks of groups using a Wilcoxon matched-pairs signed-ranks test.
(3) Compared global categorical ratings using a Friedman χ^2^ test.

To explore quantitatively the factors influencing staff and family proxy ratings, we used a linear mixed-effect regression model with three levels [(1) resident, (2) staff proxy and (3) care home] to account for clustering at care home cluster and staff level. We created two models to explore factors associated with staff and family separately so that we could explore factors specific to proxy role. We omitted demographic characteristics which overlapped.

We used NVivo software for qualitative data analysis and took a thematic analytic approach (Braun and Clarke, 2006; Nvivo, 2012). SR and a second, independent rater (KL) systematically coded the transcripts into meaningful fragments and labelled these initial codes. Discrepancies were discussed and resolved. SR, KL and PR then organised the data into preliminary themes. We discussed the coding frames within the team using a constant comparison method of coding and analysing data through three stages: open coding (examining, comparing and categorising data); axial coding (reassembling data into groupings based on thematic relationships); and selective coding (identifying the central phenomenon in the data) (Strauss and Corbin, 1998; Starks and Trinidad, 2007).

## Results

### Quantitative

#### Study participation

In total, 86/114 (75.4%) care homes contacted agreed to participate. The sample comprised 97 clusters (79 single cluster homes and 7 homes with *a* > 1 cluster, totalling 18 clusters). Of these care homes, 39 provided personal care, 13 provided nursing care and 45 provided nursing and personal care. Seventy-eight homes were privately managed, 13 were managed by a charity, four by the council, one by a not for profit organisation and one by a Local Authority Trading Company. Forty-two (43%) were dementia specialist homes. The median number of resident places in a care home cluster was 38 (IQR 27, 54).

**Fig. 1.**
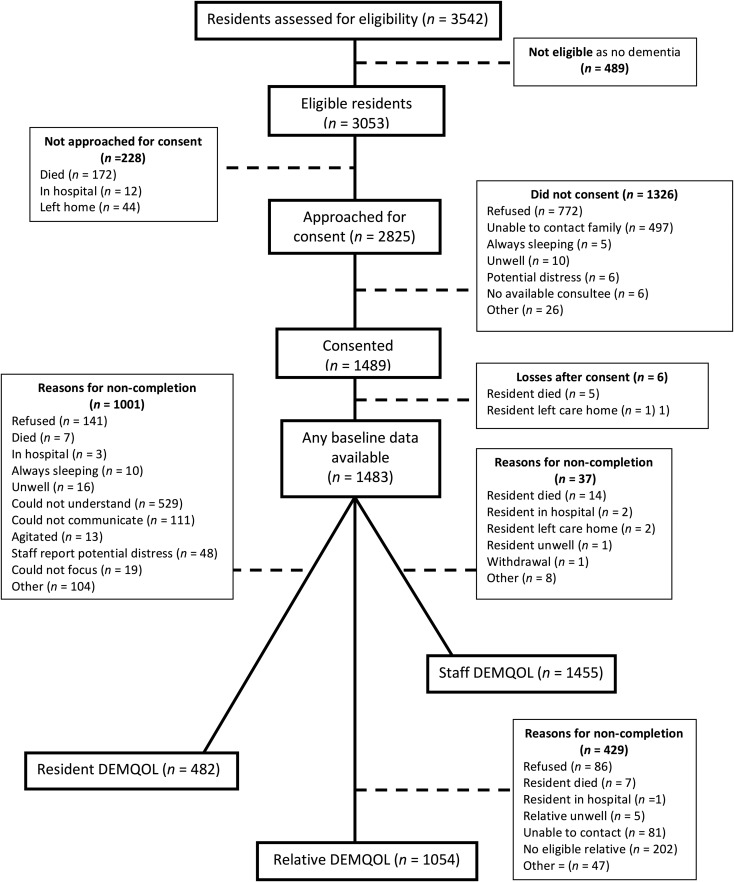
Recruitment flow chart.

Figure 1 shows resident recruitment to the study. After considering pre-existing clinical dementia diagnoses and those who screened positive on the NPC, 3053 (86.2%) residents within the care homes had probable dementia and thus were eligible. We received permission to approach 2825 residents during the recruitment phase, 1489 (52.7%) consented. Of the participating residents, 300 (20.1%) had capacity to consent to the study; we used a consultee for the remainder. Out of 1483, 1281 (86.4%) had a clinical diagnosis of dementia and the remainder had probable dementia indicated by the NPC. The number of recruited residents per cluster ranged from 2 to 55 (median 14). Participant characteristics are displayed in Table 1.

**Table 1.** Participant characteristics

Resident characteristics	
Male *n* = 1483	457 (31%)
Age, years: mean (s.d.) *n* = 1437	85 (9)
Ethnicity *n* = 1452	
White British, Irish	1324 (91%)
White other	50 (3%)
Black British, Caribbean, African, mixed	34 (2.1%)
Asian or Asian British, Indian Pakistani, Bangladeshi	13 (1%)
Chinese	2 (0.1%)
Other	29 (2%)
First language not English (*n* = 1696)	525 (31%)
Dementia severity (CDR) *n* = 1458	
Very mild	114 (8%)
Mild	313 (21%)
Moderate	482 (33%)
Severe	549 (38%)
CMAI: median, IQR	41 (33, 55)
Neuropsychiatric score: median, IQR	9 (3, 20)
Staff DEMQOL-Proxy: median, IQR	104 (95, 110)
Relative DEMQOL-Proxy: median, IQR	101 (90, 109)
Family member characteristics	
Male *n* = 1102	341 (31%)
Age, years: mean (s.d.) *n* = 1048	63 (11)
Relationship *n* = 1101	
Spouse	209 (19%)
Son or daughter	674 (61%)
Son or daughter-in-law	28 (3%)
Grandchild	15 (1%)
Friend	38 (3%)
Other	137 (12%)
Number of family visits per month: median (IQR) *n* = 1243	6 (3, 13)
Staff characteristics	
Male (*n* = 1706)	236 (14%)
Age, years: mean (s.d.)	40 (13)
Ethnicity *n* = 1480	
White British, Irish	979 (66%)
White other	151 (10%)
Chinese	8 (1%)
Black or Black British Caribbean, African, other or mixed	207 (14%)
Asian or Asian British Indian, Pakistani, Bangladeshi	120 (7%)
Mixed and other	37 (2%)

### DEMQOL-Proxy scores

**Fig. 2.**
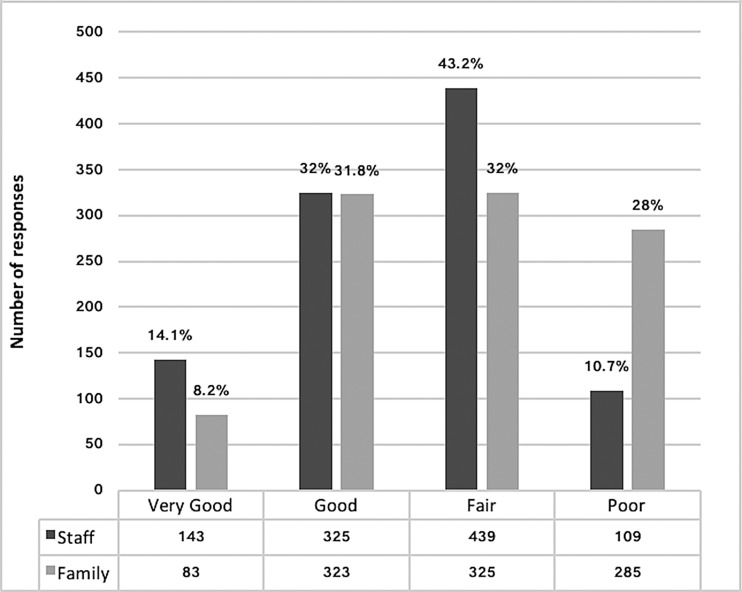
Global Ratings of Quality of Life.

The median total proxy score of residents’ QOL for staff raters was 104 (*n* = 1455, IQR 95, 110) and 101 (*n* = 1054, IQR 90, 109) for family raters (*p* < 0.001). There was a weak correlation between total scores for staff and family DEMQOL-Proxy ratings [*n* = 1054 pairs, Spearman's rho (*ρ*_s_) = 0.35]. Global ratings differed significantly between these rater groups (*n* = 1016, *p* < 0.001) (Fig. 2) with 28% of family rating QOL poor compared with 10.7% of staff and 8.2% of family rating it as very good compared with 14.1% of staff.

#### Factors associated

Regression analyses are in Table 2. Three factors were associated with a better QOL rating by *both staff and family* proxies: lower total NPI and CMAI scores and dementia severity. Higher *staff-rated* QOL was associated with: first language of the staff member rater (English) and lower ratios of staff to residents. *Higher relative-rated* QOL was associated with: being a spouse compared with being a child or other relation; resident's first language (English); and longer duration of residence in the care home.

**Table 2.** Multivariable associations of care home, resident and proxy factors with quality of life

	Staff *N* = 643		Relatives *N* = 892	
	Coefficient	95% CI	Coefficient	95% CI
Number of residents in the home	0.04	−0.04 to 0.11	−0.02	−0.07 to 0.04
Management type				
Privately managed	Reference	Reference	Reference	Reference
Other	−0.37	−3.72 to 2.98	−1.21	−4.13 to 1.71
Care home type				
Nursing	Reference	Reference	Reference	Reference
Personal care	2.22	−2.46 to 6.90	−0.62	−4.53 to 3.29
Nursing and personal care	0.38	−3.94 to 4.69	−1.67	−5.19 to 1.84
CQC ratings				
All standards met	Reference	Reference	Reference	Reference
Not all standards met	−3.60	−8.60 to 1.39	−1.89	−6.46 to 2.67
Staff resident ratio	**−0.70**	**−1.18 to −0.23**	0.06	−0.58 to 0.70
TESS score	0.00	−0.01 to 0.01	0.00	−0.01 to 0.01
Resident age	−0.05	−0.14 to 0.05	−0.06	−0.19 to 0.06
Resident sex				
Female	Reference	Reference	Reference	Reference
Male	−1.38	−3.14 to 0.38	−1.28	−3.38 to 0.82,
Resident first language English				
No	Reference	Reference	Reference	Reference
Yes	−0.85	−4.57 to 2.87	4.96	0.82 to 9.10
Clinical dementia rating				
Very mild	Reference	Reference	Reference	Reference
Mild	−0.30	−3.57 to 2.97	1.95	−2.00 to 5.91
Moderate	−0.72	−3.93 to 2.48	1.64	−2.18 to 5.46
Severe	**4.27**	**0.96 to 7.57**	**4.74**	**0.89 to 8.59**
Neuropsychiatric inventory	**−0.21**	**−0.27 to −0.14**	**−0.11**	**−0.19 to −0.04**
Agitation inventory	**−0.13**	**−0.19 to −0.07**	**−0.10**	**−0.16 to −0.04**
Length of stay	0.30	−0.03 to 0.63	**0.80**	**0.40 to 1.21**
Hospital admission				
No	Reference	Reference	Reference	Reference
Yes	−1.13	−4.37 to 2.11	−2.89	−5.96 to 0.18
Proxy sex				
Female	Reference	Reference	−0.88	−2.82 to 1.06
Male	−3.06	−6.42 to 0.32	Reference	Reference
Proxy age	0.02	−0.10 to 0.32	**–**	**–**
Relationship				
Spouse/partner	–	–	Reference	Reference
Child			**−4.63**	**−7.47 to −1.80**
Other			**−3.45**	**−6.80 to −0.10**
Frequency of visit	–	–	−0.09	−0.21 to 0.02
Months working in care homes	0.00	−0.03 to 0.03	–	–
Months working in this care home	−0.01	−0.02 to 0.01	–	–
First language English				
No	Reference	Reference	–	–
Yes	**4.02**	**1.29 to 6.76**		
Nursing qualification				
No	Reference	Reference	–	–
Yes	2.71	−0.99 to 6.40		
Constant	110.57	97.94 to 123.17	108.45	95.35 to 121.56

Significant values are in bold.

### Qualitative interviews

Twelve staff and 12 relatives were recruited from seven care homes (one nursing, six residential; six private, one charity). We interviewed four care assistants, three nurses, three senior carers and one manager: 9/12 of the staff interviewed were women; 9/12 spoke English as their first language. Their median age was 39.5 years (IQR 29.3, 46.8) and the median duration of work in care homes was 5.6 years (IQR 1.9, 9.7). Of the relatives interviewed, 8/12 were women, 10 were White British. Seven were the child or child-in-law of the care recipient, three the spouse, two were other relatives; their median age was 60 years (52.3, 71.5) and on average they visited their relatives once a week (range: every other day to monthly).

### Conceptualising QOL

We identified two main themes that influenced proxy decisions on how to rate QOL.

#### QOL = quality of care

For staff, resident's QOL was often equated with quality of care. Some staff stated that quality of care was the most important component of QOL:

I'd say his quality of health, is not very good. But when it comes to the care being given, I see that as what makes him have a good quality of life.

Female Care Assistant 1

Staff were more likely to speak about their role in enabling a good QOL.

But as staff working with her, giving her good quality of life, I actually think of her to have a good quality of life.

Female Care Assistant 2

On reflecting on filling out the DEMQOL-Proxy, one nurse highlighted the responsibility they felt in the provision of care that directly influenced QOL:

It was hard [DEMQOL-Proxy] because I want to say everyone's got an amazing quality of life here. I think it's upsetting as a carer to think that someone hasn't here, because you think is it something I'm doing? Is it something that the home's doing?

Female Nurse 1

Relatives, however, were more likely to draw an explicit distinction between QOL and quality of care:

They [staff] think he is being looked after so well so he must be all right. I don't think he is. I just said, hang on a minute, aren't we confusing quality of care with quality of life?

Son 1

Similarly, they were more likely to think about alternative care environments and consider an ‘ideal world’:

She gets a very good level of care. I am sure she would say it's [QOL] fair, to get the top box tick [Very Good] she would probably be wanting to live with me or my sister. I think fair is as good as we'd get.

Daughter 1

#### Comparing the past to the present

Relatives compared the resident's current QOL to their knowledge of how they had been before care home admission. They framed current QOL in terms of what they felt the person had lost, for example, autonomy and abilities to make choices and care for themselves.

It's very hard because when I know what he used to be and what he is now, I think. he has no life at all.

Niece 1

Relative's perceptions were also influenced by abstract judgements of what their relative would say if they could see themselves now. For some, having a worse QOL was an inevitable consequence of being in a care home because their relative would not have wanted to be there.

Before he married me, he said to me ‘. Don't put me into a care home, I would rather to beg God to take me more than you put me into a care home’. To know that now he doesn't have the capacity to say and I made that decision.

Wife 1

Life was often compared to a time before dementia and comparisons centred on what had been lost through changes caused by dementia. Accompanying these changes was a loss of their own:

Because I've lost my mum. My mum isn't there anymore. There's another person there and I still love her as my mum but it isn't my mum she's just been left to stagnate.

Daughter 2

There is a difference in his responses. to me, who is almost like a stranger at times.

Wife 2

Staff were more likely to use their understanding of dementia and focus on the disease progression:

She's got the dementia where it's at the frontal lobe. That's what causes the anger.

Female Care Assistant 3

Where relatives had come to terms with these changes and focused on the present they evaluated QOL as better:

I never dreamt, when my mother went into the care home. I didn't ever expect to see her dancing around and singing with some of the carers. It just goes to show you if you know how to help someone with dementia they can have a good quality of life.

Daughter 3

## Discussion

### Is the difference found clinically meaningful?

There were similarities and differences in how staff and family proxy raters in this sample understood a resident's QOL. Proxy ratings were weakly correlated, with staff rating resident QOL, on average, three points higher than relatives. Defining a clinically meaningful difference in QOL is highly problematic (Hays and Woolley, 2000). In most circumstances, half a standard deviation is the threshold for clinically meaningful discrimination for changes in QOL (Norman *et al*., 2003). In this study, half a deviation would be six points, which suggests that a three-point difference is not clinically meaningful. However, our finding that staff were significantly more likely to rate a resident's QOL as ‘Very Good’ whereas family members were more likely to rate it ‘Poor’ suggests that the difference may be meaningful to the proxy-raters.

### What are the factors associated with ratings?

Both staff and family proxies rated QOL higher in residents who had fewer neuropsychiatric symptoms and less agitation. These should continue to be targeted as a strategy for improving QOL (Billig, 1986; *Beer et al., 2010*; Goyder *et al*., 2012; Clare *et al*., 2014; Livingston *et al*., 2017). These factors were more strongly associated with staff than family QOL ratings. It may be that staff are more aware of or more influenced by a resident's distress and agitation, which frequently occurs during personal care.

Staff and family were more likely to rate QOL higher when the resident had more severe dementia. Perhaps they judged that those who had greater insight into their dependency had a worse QOL. Lower staff-rated QOL was associated with a higher staff to resident ratio. This may seem counter-intuitive as higher staff-rated QOL has been associated with more staff being involved in the care of each resident (Zimmerman *et al*., 2005). More staff may, however, be employed when residents have higher needs including neuropsychiatric symptoms.

Staff who spoke English as their first language tended to rate resident QOL higher, mirroring findings in a Welsh care home study (Clare *et al*., 2014). It could be that staff who share a native language with residents find it easier to communicate, understand what residents need and build relationships. Perhaps staff also found it easier to meet the needs of residents with more severe dementia and fewer neuropsychiatric symptoms positively influencing perceived QOL.

Relatives were more likely to rate QOL as higher when residents spoke English as a first language. Non-native English-speaking residents are more likely to develop problems in communicating (Hyltenstam and Stroud, 1989; Ekman *et al*., 1994; Mendez *et al*., 1999; McMurtray *et al*., 2009; Plejert *et al*., 2015; Strandroos and Antelius, 2017) and are more likely to experience agitation and this could be due to increased isolation (Cooper *et al*., 2017). This finding suggests we need to consider language in care homes given the growing cultural diversity of residents and staff in many high-income countries (Xiao *et al*., 2017).

Being a child or other family carer was associated with lower family ratings of QOL. Perhaps spouses have a more positive perception or are better able to enable the person with dementia to have a higher QOL than other carers (Novella *et al*., 2001; Conde-Sala *et al*., 2010). This difference may also relate to comparisons of the past to present. Spouses of care home residents are more likely to live with a person with dementia up until entry to the care home, when it is likely they were experiencing significant difficulties living with dementia in the community, while children or other family members may be comparing perceived QOL now to more distant memories and constructed parental identities. Carers that are not spouses may be experiencing a greater shift in the dynamic of their relationship, a continued feeling of loss, and the anxiety that they may become affected by the disease (Kjällman-Alm *et al*., 2013). The negative perception of adult children has been associated with greater carer burden (Conde-Sala *et al*., 2010) and there may be a link between carer QOL and perceived QOL (Farina *et al*., 2017).

Relatives tended to rate QOL as higher if the resident had lived longer in a care home, considering illness characteristics. This may be an indicator of a resident's adjustment (Custers *et al*., 2012; Brownie *et al*., 2014; WHO, 2015). It may also relate to the family carer's acceptance of placement, as for some QOL was negated by care home residency. Making decisions about care home placement can be stressful (Elliott *et al*., 2009; Livingston *et al*., 2010; Lord *et al*., 2015, 2016). Conflict around this decision may influence perceived QOL after placement.

### Why do staff and family think differently about QOL?

Whilst staff and family agree on some important factors contributing to a good QOL, they have different experiences of the resident and a resident may present differently when relatives visit. They also have different relationships and roles in the resident's life.

Relative's longstanding personal relationships are accompanied by past experiences, negotiated attachments and internal emotional processes. Family members’ prior knowledge of an individual may be used in their judgement about QOL: comparing how they were and what they wanted to the reality now. Relatives that rated proxy QOL higher were more likely to describe focusing on the present when making proxy-ratings and to describe an acceptance of care home placement and the progression of dementia.

In contrast, staff have a professional role, ascribed purpose and perceived value in the resident's QOL. Perceiving an active role in the provision of QOL is important to find meaning in caring. Care staff are often disempowered and undervalued. Finding meaning and having intrinsic motivations in caring is associated with higher caregiving satisfaction (Lyonette and Yardley, 2003; Quinn *et al*., 2012a, 2012b). Relatives might struggle to find a meaningful role in a care home and feelings of powerlessness may negatively impact their wellbeing (Quinn *et al*., 2015). Family carer QOL is poorer when people with dementia lived in a care home (Argimon *et al*., 2005; Reidijk *et al*., 2006; Farina *et al*., 2017).

A proxy rater's understanding of dementia may also influence how they make sense of a person's lived experience in the present. Illness representations held by family members of people living with dementia influence their understanding of what is happening to the person and how they respond and provide support (Quinn *et al*., 2017). Family are less likely than staff to receive training on dementia. Educating family members about dementia and acceptance and emotion-focused coping can reduce the affective symptoms and case-level depression of carers of family members with dementia (Livingston *et al*., 2014b) and may improve family proxy raters’ perceptions of QOL.

### Strength and limitations

Whilst we studied a large and diverse sample of care homes, these were not recruited randomly. Better resourced care homes may be more able to accommodate research. This correlation between staff and family proxy ratings is weaker than previously reported (Beer *et al*., 2010; Clare *et al*., 2014; Robertson *et al*., 2017) which may be due to a difference in QOL measures. It could be that the indicators provided in the DEMQOL-Proxy are more sensitive to capturing differences in the perspectives between proxies (Jing *et al*., 2016). It may be that the DEMQOL-Proxy itself shaped participant responses in interview but we left time in between quantitative and qualitative responses lessen this impact. The study uses the DEMQOL-Proxy which restricts answers to predefined questions pertaining to an individual's feelings and how worried an individual has been about their memory, everyday life. In this study, our understanding is enriched by broad, holistic global judgements in the qualitative analysis.

## Conclusions

Proxy ratings are influenced by the rater's own context and experience of caring. While all raters reported higher QOL when the resident had fewer neuropsychiatric symptoms, staff judged a resident's QOL to be significantly higher than family members did. We found that staff were more likely to view QOL as synonymous with ‘quality of care’. Relatives, however, had a longstanding personal relationship with the resident, their own fears, understanding and sense of loss for themselves and their relative influenced their judgement of QOL. Some relatives felt it was impossible to have a good QOL whilst living in a care home.

## Implications

Ratings of QOL are subjective outcomes; especially when considering how the person with dementia feels. Staff and family proxy ratings cannot be used interchangeably. If future studies include proxy-rated QOL as a variable and use a mixture of staff and proxy raters, analyses must control for the status of the proxy rater. Proxy ratings may offer a unique insight to perceived QOL and the contributing factors and may be an important target for improving carer QOL. Psychological interventions that promote education and focus on acceptance may benefit family carers as well as systemic interventions that promote inclusion, find roles for relatives and support staff and relatives to understand each other's perspective and communicate. The only factors identified with both staff and family perspectives are the resident's mental health and agitation which should remain targets for interventions to improve QOL, enabling people to live well with dementia. Research should consider these findings when evaluating the success of interventions.

## References

[ref51] Argimon JM, Limon E, Vila J and Cabezas C (2005) Health-related quality-of- life of care-givers as a predictor of nursing-home placement of patients with dementia. Alzheimer Disease Associated Disorders 19, 41–44. 15764871 10.1097/01.wad.0000160343.96562.8e

[ref52] Banerjee S, Samsi K, Petrie CD, Alvir J, Treglia M, Schwam EM and del Valle M (2009) What do we know about quality of life in dementia? A review of the emerging evidence on the predictive and explanatory value of disease specific measures of health related quality of life in people with dementia. International Journal of Geriatric Psychiatry 24, 15–24. 18727132 10.1002/gps.2090

[ref1] Beer C, Flicker L, Horner B, Bretland N, Scherer S, Lautenschlager NT, Shaper F and Almeida OP (2010) Factors associated with self and informant ratings of the quality of life of people with dementia living in care facilities: a cross sectional study. PLoS ONE 5, e15621. doi: 10.1371/journal.pone.0015621. 21179448 PMC3001486

[ref2] Beerens HC, Sutcliffe C, Renom-Guiteras A, Soto ME, Suhonen R, Zabalegui A, Bökberg C, Saks K and Hamers JP and RightTimePlaceCare Consortium (2014) Quality of life and quality of care for people with dementia receiving long term institutional care or professional home care: the European righttimeplacecare study. Journal American Medical Directors Association 15, 54–61. 10.1016/j.jamda.2013.09.010 24220139

[ref3] Billig N (1986) Agitated behaviors in the elderly: i. *A conceptual review*. Journal American Geriatric Society 34, 711–721. 10.1111/j.1532-5415.1986.tb04302.x 3531296

[ref4] Braun V and Clarke V (2006) Using thematic analysis in psychology. Qualitative Research in Psychology 3, 77–101.

[ref5] Brownie S, Horstmanshof L and Garbutt R (2014) Factors that impact residents’ transition and psychological adjustment to long-term aged care: a systematic literature review. International Journal of Nursing Studies 51, 1654–1666. 24813582 10.1016/j.ijnurstu.2014.04.011

[ref6] Challis D, Mozley CG, Sutcliffe C, Bagley H, Price L, Burns A, Huxley P and Cordingley L (2000) Dependency in older people recently admitted to care homes. Age and Ageing 29, 255–260. 10855909 10.1093/ageing/29.3.255

[ref7] Chua KC, Brown A, Little R, Matthews D, Morton L, Loftus V, Watchurst C, Tait R, Romeo R and Banerjee S (2016) Quality-of-life assessment in dementia: the use of DEMQOL and DEMQOL-proxy total scores. Quality of Life Research 25, 3107–3118. 27318488 10.1007/s11136-016-1343-1 PMC5102947

[ref8] Clare L, Quinn C, Hoare Z, Whitaker R and Woods RT (2014) Care staff and family member perspectives on quality of life in people with very severe dementia in long-term care: a cross-sectional study. Health Quality Life Outcomes 12, 175. 10.1186/s12955-014-0175-3 PMC4276099 25488722

[ref53] Cohen-Mansfield J and Billig N (1986) Agitated behaviours in the elderly. A conceptual review. Journal American Geriatric Society 38 (10), 711–721. 10.1111/j.1532-5415.1986.tb04302.x 3531296

[ref9] Conde-Sala JL, Garre-Olmo J, Turró-Garriga O, Vilalta-Franch J and López-Pousa S (2010) Quality of life of patients with Alzheimer's disease: differential perceptions between spouse and adult child caregivers. Dementia and Geriatric Cognitive Disorders 29, 97–108. 20150730 10.1159/000272423

[ref10] Cooper C, Rapaport P, Robertson S, Marston L, Barber J, Manela M and Livingston G (2017) Relationship between speaking English as a second language and agitation in people with dementia living in care homes: results from the MARQUE (Managing Agitation and Raising Quality of life) English national care home survey. International Journal of Geriatric Psychiatry 33, 504–509. 28971511 10.1002/gps.4786 PMC5836957

[ref11] Cummings JL, Mega M, Gray K, Rosenberg-Thompson S, Carusi DA and Gornbein J (1994) The neuropsychiatric inventory: comprehensive assessment of psychopathology in dementia. Neurology 44, 2308–2308. 7991117 10.1212/wnl.44.12.2308

[ref12] Custers AFJ, Westerhof GJ, Kuin Y, Gerritsen DL and Riksen-Walraven JM (2012) Relatedness, autonomy, and competence in the caring relationship: the perspective of nursing home residents. Journal of Aging Studies 26, 319–326.

[ref13] Ekman SL, Wahlin TBR, Norberg A, Viitanen M and Winblad B (1994) Preconditions for communication in the care of bilingual demented persons. International Psychogeriatrics 6, 105–120. 8054490 10.1017/s1041610294001675

[ref14] Elliott BA, Gessert CE and Peden-Mcalpine C (2009) Family decision-making in advanced dementia: narrative and ethics. Scandinavian Journal of Caring Sciences 23, 251–258. 19645802 10.1111/j.1471-6712.2008.00613.x

[ref15] Farina N, Page TE, Daley S, Brown A, Bowling A, Basset T, Livingston G, Knapp M, Murray J and Banerjee S (2017) Factors associated with the quality of life of family carers of people with dementia: a systematic review. Alzheimer's & Dementia 13, 572–581. 10.1016/j.jalz.2016.12.010 28167069

[ref16] Goyder J, Orrell M, Wenborn J and Spector A (2012) Staff training using STAR: a pilot study in UK care homes. International Psychogeriatrics 24, 911–920. 22217445 10.1017/S1041610211002559

[ref17] Gräske J, Fischer T, Kuhlmey A and Wolf-Ostermann K (2012) Quality of life in dementia care – differences in quality of life measurements performed by residents with dementia and by nursing staff. Aging and Mental Health 16, 819–827. 22486538 10.1080/13607863.2012.667782

[ref18] Hays RD and Woolley JM (2000) The concept of clinically meaningful difference in health-related quality-of-life research: how meaningful is it? Pharmacoeconomics 18, 419–423. 11151395 10.2165/00019053-200018050-00001

[ref19] Hoe J, Hancock G, Livingston G and Orrell M (2006) Quality of life of people with dementia in residential care homes. British Journal of Psychiatry 188, 460–464. 16648533 10.1192/bjp.bp.104.007658

[ref20] Hughes CP, Berg L, Danziger WL, Coben LA and Martin RL (1982) A new clinical scale for the staging of dementia. British Journal of Psychiatry 140, 566–572. 7104545 10.1192/bjp.140.6.566

[ref21] Hyltenstam K and Stroud C (1989) Bilingualism in Alzheimer's dementia: two case studies In Hyltenstam K and Obler K (eds), Bilingualism Across the Lifespan: Aspects of Acquisition, Maturity and Loss. Cambridge: Cambridge University Press, pp. 202‒226.

[ref22] Jing W, Willis R and Feng Z (2016) Factors influencing quality of life of elderly people with dementia and care implications: a systematic review. Archives of Gerontology and Geriatrics 66, 23–41. 27176488 10.1016/j.archger.2016.04.009

[ref23] Kjällman-Alm A, Norbergh KG and Hellzen O (2013) What it means to be an adult child of a person with dementia. International Journal of Qualitative Studies on Health and Well-Being 8, 21676. doi: 10.3402/qhw.v8i0.21676 24152431 PMC3807013

[ref54] Levin E (1989) *Noticeable Problems Checklist*. National Institute for Social Work, London.

[ref26] Livingston G, Leavey G, Manela M, Livingston D, Rait G, Sampson E, Bavishi S, Shahriyarmolki K and Cooper C (2010) Making decisions for people with dementia who lack capacity: qualitative study of family carers in UK. The British Medical Journal 341, 494. 10.1136/bmj.c4184 PMC2923693 20719843

[ref55] Livingston G, Cooper C, Woods J, Milne A and Katona C (2014a) Successfully ageing in adversity: the LASER-AD longitudinal study. Journal of Neurology, Neurosurgery & Psychiatry 79, 641–645. 10.1136/jnnp.2007.126706 17898031

[ref56] Livingston G, Barber J, Rapaport P, Knapp M, Griffin M, Romeo R, King D, Livingston D, Lewis-Holmes E, Mummery C, Walker Z, Hoe J and Cooper C (2014b) START (STrAtegies for RelaTives) study: a pragmatic randomised controlled trial to determine the clinical effectiveness and cost-effectiveness of a manual-based coping strategy programme in promoting the mental health of carers of people with dementia. Health Technology Assessment 18, 1–242. 10.3310/hta18610 PMC4781467 25300037

[ref27] Livingston G, Barber J, Marston L, Rapaport P, Livingston D, Cousins S, Robertson S, La Frenais F and Cooper C (2017) Prevalence of and associations with agitation in residents with dementia living in care homes: MARQUE cross-sectional study. The British Journal of Psychiatry 3, 171–178. 10.1192/bjpo.bp.117.005181 PMC5530006 28794896

[ref28] Lord K, Livingston G and Cooper C (2015) A systematic review of barriers and facilitators to and interventions for proxy decision-making by family carers of people with dementia. International Psychogeriatrics 27, 1301–1312. 25870004 10.1017/S1041610215000411

[ref29] Lord K, Livingston G, Robertson S and Cooper C (2016) How people with dementia and their families decide about moving to a care home and support their needs: development of a decision aid, a qualitative study. BMC Geriatrics 16, 68. doi: 10.1186/s12877-016-0242-1. 27001704 PMC4802590

[ref57] Lyonette C and Yardley L (2003). The influence on carer wellbeing of motivations to care for older people and the relationship with the care recipient. Ageing and Society 23, 487–506.

[ref30] Magaziner J (1997) Use of proxies to measure health and functional outcomes in effectiveness research in persons with Alzheimer disease and related disorders. Alzheimer's Disease & Associated Disorders 11, 168–174. 9437462

[ref31] McMurtray A, Saito E and Nakamoto B (2009) Language preference and development of dementia among bilingual individuals. Hawaii Medical Journal 68, 223–226. 19842364 PMC4335728

[ref32] Mendez MF, Perryman KM, Pontón MO and Cummings JL (1999) Bilingualism and dementia. The Journal of Neuropsychiatry & Clinical Neurosciences 11, 411–412. 10440021 10.1176/jnp.11.3.411

[ref33] Moriarty J and Webb S (2000) Part of Their Lives – Community Care for Older People with Dementia. Bristol, UK: The Policy Press.

[ref34] Norman GR, Sloan JA and Wyrwich KW (2003) Interpretation of changes in health-related quality of life the remarkable universality of half a standard deviation. Medical Care 41, 582–592. 12719681 10.1097/01.MLR.0000062554.74615.4C

[ref35] Novella JL, Jochum C, Jolly D, Morrone I, Ankri J, Bureau F and Blanchard F (2001) Agreement between patients’ and proxies’ reports of quality of life in Alzheimer's disease. Quality of Life Research 10, 443–452. 11763206 10.1023/a:1012522013817

[ref36] NVivo (2012) Qualitative data analysis software; QSR International Pty Ltd. Version 10.

[ref37] O'Rourke HM, Fraser KD and Duggleby W (2015) Does the quality of life construct as illustrated in quantitative measurement tools reflect the perspective of people with dementia? Journal of Advance Nursing 71, 1812–1824. 10.1111/jan.12667 25892121

[ref38] Perales J, Cosco TD, Stephan BCM, Haro JM and Brayne C (2013) Health-related quality-of-life instruments for Alzheimer's disease and mixed dementia. International Psychogeriatrics 25, 691–706. 23347698 10.1017/S1041610212002293

[ref39] Plejert C, Antelius E, Yazdanpanah M and Nielsen TR (2015) ‘There's a letter called ef’ on challenges and repair in interpreter-mediated tests of cognitive functioning in dementia evaluations: a case study. Journal of Cross-Cultural Gerontology 30, 163–187. 25982531 10.1007/s10823-015-9262-0

[ref58] Quinn C, Clare L, McGuinness T and Woods R (2012a) The impact of relationships, motivations, and meanings on dementia caregiving outcomes. International Psychogeriatrics 24, 1816–1826. 22652014 10.1017/S1041610212000889

[ref59] Quinn C, Clare L and Woods RT (2012b). What predicts whether caregivers of people with dementia find meaning in their role? International Journal of Geriatric Psychiatry 27, 1195–1202. 22334416 10.1002/gps.3773

[ref60] Quinn C, Clare L, Jelley H, Bruce E and Woods B (2014) ‘It's in the eyes’: how family members and care staff understand awareness in people with severe dementia. Aging Mental Health 18, 260–268. 23984831 10.1080/13607863.2013.827627

[ref61] Quinn C, Clare L and Woods R (2015) Balancing needs: the role of motivations, meanings and relationship dynamics in the experience of informal caregivers of people with dementia. Dementia 12, 220–237. 10.1177/1471301213495863 24339101

[ref62] Quinn C, Jones IR and Clare L (2017) Illness representations in caregivers of people with dementia. Aging & Mental Health 21, 553–561. 26729580 10.1080/13607863.2015.1128882

[ref63] Riedijk SR, De Vugt ME, Duivenvoorden HJ, Niermeijer MF, Van Swieten JC, Verhey FR and Tibben A (2006) Caregiver burden, health-related quality of life and coping in dementia caregivers: a comparison of frontotemporal dementia and Alzheimer's disease. Dementia Geriatric Cognitive Disorder 22, 405–412. 10.1159/000095750 16966830

[ref40] Robertson S, Cooper C, Hoe J, Hamilton O, Stringer A and Livingston G (2017) Proxy rated quality of life of care home residents with dementia: a systematic review. International Psychogeriatrics 29, 569–581. 28088926 10.1017/S1041610216002167 PMC5964456

[ref41] Sloane PD, Mitchell CM, Weisman G, Zimmerman S, Foley KML, Lynn M, Calkins M, Lawton MP, Teresi J, Grant L, Lindeman D and Montgomery R (2002) The Therapeutic Environment Screening Survey for Nursing Homes (TESS-NH): an observational instrument for assessing the physical environment of institutional settings for persons with dementia. The Journals of Gerontology. Series B, Psychological Sciences and Social Sciences 57, 69–78. 10.1093/geronb/57.2.s69 11867668

[ref64] Smith SC, Lamping DL, Banerjee S, Harwood R, Foley B, Levin E, Mann A and Knapp M (2005) Measurement of health-related quality of life for people with dementia: development of a new instrument (DEMQOL) and an evaluation of current methodology. Health technology Assessment, 9 (10). 10.3310/hta9100. 15774233

[ref43] Smith SC, Lamping DL, Banerjee S, Harwood RH, Foley B, Smith P, Cook JC, Murray J, Prince M, Levin E, Mann A and Knapps M (2007) Development of a new measure of health-related quality of life for people with dementia: DEMQOL. Psychological Medicine 37, 737–746. 17176501 10.1017/S0033291706009469

[ref44] Starks H and Trinidad SB (2007) Choose your method: a comparison of phenomenology, discourse analysis, and grounded theory. Qualitative Health Research 10, 1372–1380. doi: 10.1177/1049732307307031. 18000076

[ref45] StataCorp (2015) Stata Statistical Software: Release 14. College Station, TX: StataCorp LP.

[ref46] Strandroos L and Antelius E (2017) Interaction and common ground in dementia: communication across linguistic and cultural diversity in a residential dementia care setting. Health *(*London*)* 21, 538–554. 27895101 10.1177/1363459316677626

[ref47] Strauss A and Corbin J (1998) Basics of Qualitative Research: Techniques and Procedures for Developing Grounded Theory, 2nd Edn Thousand Oaks, CA: Sage.

[ref48] World Health Organization (2015) World Report on Ageing and Health. Geneva: WHO Available at http://apps.who.int/iris/bitstream/handle/10665/186463/9789240694811_eng.pdf;jsessionid=FD10C9B3FD26BB80299A0037843EB321?sequence=1 (Accessed 5 December 2018).

[ref49] Xiao LD, Willis E, Harrington A, Gillham D, De Bellis A, Morey W and Jeffers L (2017) Resident and family member perceptions of cultural diversity in aged care homes. Nursing & Health Sciences 19, 59–65. 27485390 10.1111/nhs.12302

[ref50] Zimmerman S, Sloane PD, Williams CS, Reed PS, Preisser JS, Eckert JK, Boustani M and Dobbs D (2005) Dementia care and quality of life in assisted living and nursing homes. The Gerontologist 45, 133–146. 16230760 10.1093/geront/45.suppl_1.133

